# Selective functional antibody transfer into the breastmilk after SARS-CoV-2 infection

**DOI:** 10.1016/j.celrep.2021.109959

**Published:** 2021-10-22

**Authors:** Krista M. Pullen, Caroline Atyeo, Ai-Ris Y. Collier, Kathryn J. Gray, Mandy B. Belfort, Douglas A. Lauffenburger, Andrea G. Edlow, Galit Alter

**Affiliations:** 1 Department of Biological Engineering, Massachusetts Institute of Technology, Cambridge, MA 02139, USA; 2 Ragon Institute of MGH, MIT, and Harvard, Cambridge, MA 02139, USA; 3 PhD Program in Virology, Division of Medical Sciences, Harvard University, Boston, MA 02115, USA; 4 Department of Obstetrics, Gynecology and Reproductive Biology, Beth Israel Deaconess Medical Center, Harvard Medical School, Boston, MA, USA; 5 Center for Virology and Vaccine Research, Beth Israel Deaconess Medical Center, Harvard Medical School, Boston, MA, USA; 6 Department of Obstetrics, Gynecology and Reproductive Biology, Brigham and Women’s Hospital, Harvard Medical School, Boston, MA 02115, USA; 7 Department of Pediatric Newborn Medicine, Brigham and Women’s Hospital, Harvard Medical School, Boston, MA 02115, USA; 8 Department of Obstetrics, Gynecology and Reproductive Biology, Massachusetts General Hospital, Harvard Medical School, Boston, MA 02114, USA; 9 Vincent Center for Reproductive Biology, Massachusetts General Hospital, Boston, MA 02114, USA

**Keywords:** COVID-19, breastmilk, pregnancy, antibody transfer, Fc-receptor, neutrophils, neutralization, SARS-CoV-2

## Abstract

Antibody transfer via breastmilk represents an evolutionary strategy to boost immunity in early life. Although severe acute respiratory syndrome coronavirus 2 (SARS-CoV-2)-specific antibodies have been observed in the breastmilk, the functional quality of these antibodies remains unclear. Here, we apply systems serology to characterize SARS-CoV-2-specific antibodies in maternal serum and breastmilk to compare the functional characteristics of antibodies in these fluids. Distinct SARS-CoV-2-specific antibody responses are observed in the serum and breastmilk of lactating individuals previously infected with SARS-CoV-2, with a more dominant transfer of immunoglobulin A (IgA) and IgM into breastmilk. Although IgGs are present in breastmilk, they are functionally attenuated. We observe preferential transfer of antibodies capable of eliciting neutrophil phagocytosis and neutralization compared to other functions, pointing to selective transfer of certain functional antibodies to breastmilk. These data highlight the preferential transfer of SARS-CoV-2-specific IgA and IgM to breastmilk, accompanied by select IgG subpopulations, positioned to create a non-pathologic but protective barrier against coronavirus disease 2019 (COVID-19).

## Highlights

• SARS-CoV-2 induces pathogen-specific antibodies in maternal blood and breastmilk
• Breastmilk antibodies are functionally attenuated compared to serum antibodies
• Neutrophil phagocytosis and neutralization are preferentially present in breastmilk
• Disease severity, but not time since symptom onset, impacts transfer to breastmilk

## Graphical abstract

**Figure:**
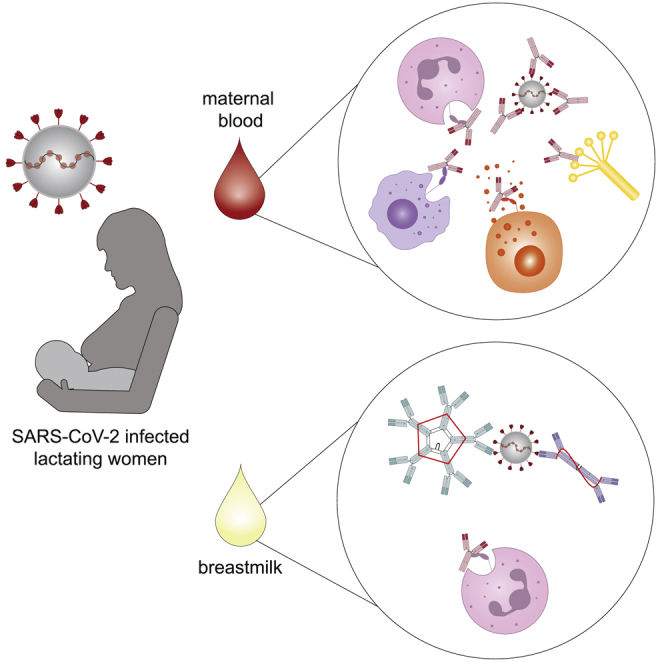

## Teaser

Pullen et al. characterize the functionality of SARS-CoV-2-specific antibodies in maternal blood and breastmilk after SARS-CoV-2 infection. Although infection elicits polyfunctional IgG in blood, breastmilk mostly contains neutralizing and neutrophil-activating IgA, IgM, and IgG, suggesting a selection of specific, non-inflammatory antibody subsets in the breastmilk.

## Introduction

The rapid spread of severe acute respiratory syndrome coronavirus 2 (SARS-CoV-2) has resulted in millions of deaths and hundreds of millions of hospitalizations (CDC, 2021). Certain populations exhibit a higher risk of developing severe disease, including individuals with pre-existing heart, respiratory, metabolic, and autoimmune conditions (Ssentongo et al., 2020; Zhou et al., 2020). Although children have been less widely impacted than adults by coronavirus disease 2019 (COVID-19) (Ludvigsson, 2020; Zimmermann and Curtis, 2020a, 2020b), infants and neonates are the most at-risk pediatric group. While hospitalization numbers of SARS-CoV-2 infants may be inflated because infants are often hospitalized to rule out sepsis (Hassan et al., 2021; Zeng et al., 2020), neonates and infants may also be more prone to severe disease upon infection with SARS-CoV-2 compared to older pediatric populations (Dong et al., 2020; Kim et al., 2020; Preston et al., 2021). This bimodal distribution of severity, with intense susceptibility in early life and then again in older adulthood, resembles that seen in other respiratory diseases, like influenza and tuberculosis (Clohisey and Baillie, 2019; Nair et al., 2011; Schaaf et al., 2010; Shingadia and Novelli, 2003). Even if the specific mechanisms that underlie this age-dependent change in respiratory pathogen susceptibility remain unclear, the early-life predisposition to severe respiratory disease points to the urgent need to develop vaccines able to rapidly drive immunity in infants.

Evolutionarily, infants receive passive immunity through the transfer of systemic antibodies via the placenta and mucosal antibodies via breastmilk (Atyeo and Alter, 2021; Langel et al., 2020). Systemic antibodies are thought to confer protection for 3–9 months (Kiliç et al., 2003; Leuridan and Van Damme, 2007; Leuridan et al., 2011; Ochola et al., 2009; Schlaudecker et al., 2013; Watanaveeradej et al., 2003), until the infant is able to mount an active immune response. Breastmilk antibodies are derived primarily from B cells primed in the mucosa, resulting in high concentrations of secretory antibodies that offer a prolonged period of immune transfer to confer immunity against mucosal pathogens. Breastfeeding offers protection against several enteric and respiratory infections, including protection from Shigella (Durand et al., 2013), influenza (Husseini et al., 1984; Schlaudecker et al., 2013), respiratory syncytial virus (Bulkow et al., 2002; Downham et al., 1976), and HIV (Fouda et al., 2011; Mabuka et al., 2012; Pollara et al., 2015). Moreover, recent data also suggest that SARS-CoV-2-specific antibodies are transferred via breastmilk to infants, potentially providing an early source of immunity to protect the infant from infection or disease (Fox et al., 2020; Pace et al., 2020). However, the precise levels and quality of the antibodies transferred is less well understood.

Breastmilk is a complex mixture rich in nutrients, cytokines, cells, and antibodies (Andreas et al., 2015; Casey et al., 1986; Lyons et al., 2020). Immunoglobulin A (IgA) is the dominant antibody transferred to infants via breastmilk, thought to play a critical role in mucosal defense by supporting commensalization (Palm et al., 2014; Rogier et al., 2014) and excluding pathogens (Binsker et al., 2020; Harris et al., 2006). However, mounting data across infectious pathogens suggest that additional antibody subpopulations, including IgG, are also transferred across the breastmilk, contributing to immune protection (Andreas et al., 2015; Caballero-Flores et al., 2019; Koch et al., 2016). Whether all antibody isotypes and subclasses transfer equally or whether preferential transfer of IgG, IgA, and other antibodies into breastmilk occurs remains incompletely understood but could provide critical insights for rational vaccine and monoclonal therapeutic design in the future to improve delivery of antibodies to neonates via breastmilk.

Thus, to better understand the mechanism of antibody transfer to breastmilk, particularly in the setting of SARS-CoV-2 infection, we used systems serology to profile antibody Fc characteristics of SARS-CoV-2-specific responses in a cohort of 45 matched maternal serum-breastmilk dyads (19 SARS-CoV-2 + and 26 SARS-CoV-2 −). A clear anti-SARS-CoV-2 response was detected in the serum and breastmilk of SARS-CoV-2-infected mothers compared to uninfected mothers, marked by a dominant IgA and IgM response in breast milk and an IgG response in serum. Whereas a polyfunctional anti-spike response was detected in serum, more limited antibody functionality was transferred to the breastmilk. These results confirm preferential transfer of spike-specific secretory IgA and IgM into the breastmilk and the presence of functionally selected IgG antibodies into the breastmilk upon SARS-CoV-2 infection, potentially as a mechanism to promote the transfer of protective but non-inflammatory antibodies into the newborn.

## Results

### SARS-CoV-2 infection in pregnancy is associated with a distinct serum and breastmilk antibody response

**Figure 1:**
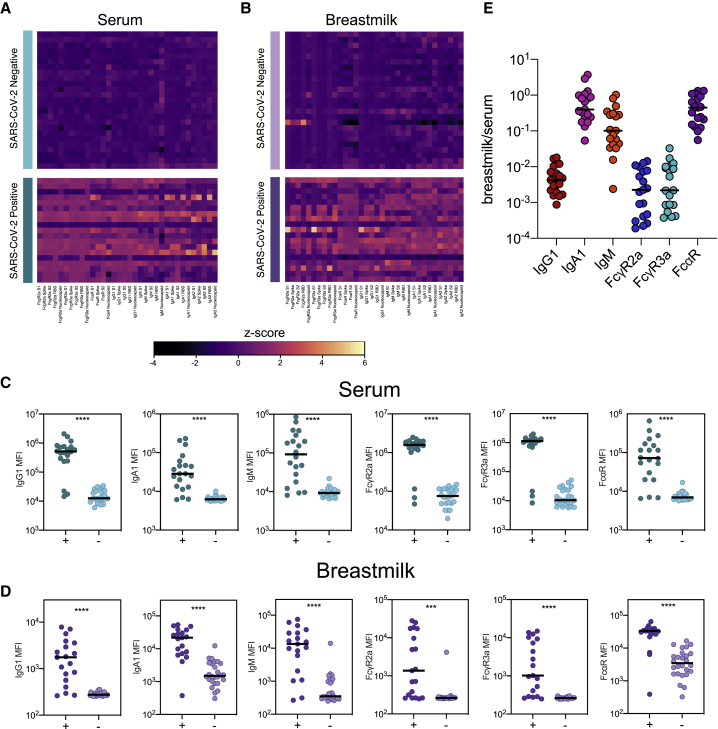
SARS-CoV-2-infected women induce an antibody response in serum and breastmilk (A and B) The heatmaps summarize the antibody isotypes and FcR-binding signatures against SARS-CoV-2 in serum (A) and breastmilk (B) for SARS-CoV-2 uninfected (top) and infected (bottom) women. The color scale corresponds with the *Z* score for each antibody titer measured, with lighter coloration representing a positive *Z* score and darker coloration representing a negative *Z* score. The data represent the average of two replicates. (C) The dot plots show the IgG1, IgA1, and IgM titers and FcgR2a, FcgR3a, and FcaR binding against SARS-CoV-2 spike in the serum of SARS-CoV-2-infected (left, dark teal) and uninfected (right, light teal) mothers. Significance was determined by Mann-Whitney test; ^∗^p < 0.05, ^∗∗^p < 0.01, ^∗∗∗^p < 0.001, and ^∗∗∗∗^p < 0.0001. The data represent the average of two replicates. (D) The dot plots show the IgG1, IgA1, and IgM titers and FcgR2a, FcgR3a, and FcaR binding against SARS-CoV-2 spike in the breastmilk from SARS-CoV-2-infected (left, dark purple) and uninfected (right, light purple) mothers. Significance was determined by Mann-Whitney test; ^∗^p < 0.05, ^∗∗^p < 0.01, ^∗∗∗^p < 0.001, and ^∗∗∗∗^p < 0.0001. The data represent the average of two replicates. (E) The dot plots show the ratio of titers and FcR-binding against SARS-CoV-2 spike in breastmilk to serum. Significance was determined by a one-way ANOVA; ^∗^p < 0.05, ^∗∗^p < 0.01, ^∗∗∗^p < 0.001, and ^∗∗∗∗^p < 0.0001.

Recent studies have demonstrated the transfer of SARS-CoV-2-specific neutralizing antibodies in breastmilk following SARS-CoV-2 infection (Fox et al., 2020; Pace et al., 2020). However, beyond binding and blocking the virus, emerging data point to a critical role for extra-neutralizing Fc-effector functions in resolution of infection and disease (Excler et al., 2014; Lu et al., 2018). Previous studies clearly illustrated the evolution and transfer of Fc-effector function in pregnant women via placenta to their infants, but less is known about the transfer of Fc-effector function across breastmilk. To better understand the Fc transfer profile of SARS-CoV-2-specific antibodies to the breastmilk, we used systems serology on samples from a cohort of 45 matched maternal serum-breastmilk dyads (Table 1). Systems serology profiling revealed that each woman possessed a unique SARS-CoV-2-specific antibody profile, with distinct signatures characterizing the serum and milk (Figures 1A and 1B). As expected, mothers infected with SARS-CoV-2 possessed SARS-CoV-2-specific antibodies in serum and breastmilk that were not present in SARS-CoV-2-uninfected specimens (Figures 1C and 1D). Univariate analysis demonstrated that, while SARS-CoV-2-specific antibody titers were observed across both compartments in COVID+ samples compared to COVID− samples, the levels of all isotypes were persistently lower in breastmilk than serum (Figures 1C–1E, S1, and S2). To further define whether particular antibody subpopulations were transferred preferentially into breastmilk, a transfer ratio was calculated for each isotype and Fc-receptor binding feature (Figure 1C). We observed robust transfer of IgA and IgM in breastmilk, with more limited IgG1 transfer (Figure 1C). Although the placenta preferentially transfers Fc-receptor (FcR) binding antibodies, at the level of the breast, we observed limited IgG and IgG-binding Fcγ-receptor (FcγR) transfer but robust IgM, IgA,and IgA-binding FcR (FcαR) transfer, supporting preferential transfer of IgA and IgM in breastmilk following SARS-CoV-2 infection in pregnant women.

**Table 1:** Demographics of cases and controls

	COVID negative (n = 26)^a^	COVID positive (n = 20)^b^
Maternal age (median, IQR)	33 (31, 38)	32 (27, 36)

**Race**		

Black	2 (8%)	3 (15%)
White	18 (69%)	15 (75%)
Other	6 (23%)	2 (10%)

**Ethnicity**		

Hispanic or Latino	4 (15%)	5 (25%)

**Neonatal sex**		

Female	13 (50%)	10 (50%)

**COVID severity**^c^		

Asymptomatic/mild	–	10 (50%)
Moderate/severe/critical	–	10 (50%)
Time from positive test to serum sample collection (median, IQR)	–	66 (11.5–99.5)
Time from symptom onset to breastmilk collection (median, IQR)		78 (15,123)

a n = 26 SARS-CoV-2 negative at delivery, never + for SARS-CoV-2 in pregnancy, and no symptoms.

b n = 20 SARS-CoV-2 positive in pregnancy. No neonates tested positive for SARS-CoV-2 by nasopharyngeal swab at delivery.

c COVID severity determined per NIH criteria

### Exclusion of particular antibody functions from breastmilk

**Figure 2:**
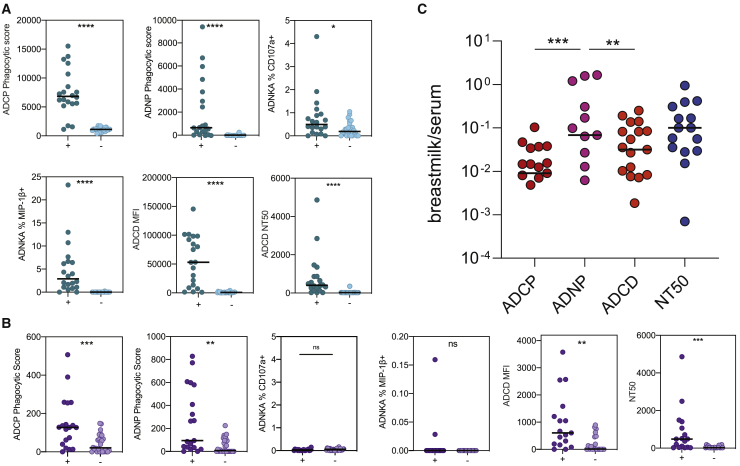
Breastmilk antibodies have limited antibody-dependent natural killer cell activation (ADNKA) (A) The dot plots show the antibody-dependent cellular phagocytosis (ADCP), antibody-dependent neutrophil phagocytosis (ADNP), antibody-dependent natural killer cell activation (ADNKA) (CD107a and MIP-1b), and antibody-dependent complement deposition (ADCD) activity against SARS-CoV-2 spike in the serum of SARS-CoV-2-infected (left, dark teal) and uninfected (right, light teal) mothers. Significance was determined by Mann-Whitney test; ^∗^p < 0.05, ^∗∗^p < 0.01, ^∗∗∗^p < 0.001, and ^∗∗∗∗^p < 0.0001. The data represent the average of two replicates (ADCP and ADCD) or two donors (ADNP and ADNKA). (B) The dot plots show the ADCP, ADNP, ADNKA (CD107a and MIP-1b), and ADCD activity against SARS-CoV-2 spike in the breastmilk from SARS-CoV-2-infected (left, dark purple) and uninfected (right, light purple) mothers. Significance was determined by Mann-Whitney test; ^∗^p < 0.05, ^∗∗^p < 0.01, ^∗∗∗^p < 0.001, and ^∗∗∗∗^p < 0.0001. The data represent the average of two replicates (ADCP and ADCD) or two donors (ADNP and ADNKA). (C) The dot plot shows the ratio of functional activity against SARS-CoV-2 spike in breastmilk to serum. Significance was determined by a one-way ANOVA; ^∗^p < 0.05, ^∗∗^p < 0.01, ^∗∗∗^p < 0.001, and ^∗∗∗∗^p < 0.0001.

Although emerging data point to the transfer of neutralizing antibodies in breastmilk (Fox et al., 2020; Pace et al., 2020), less is known about the extra-neutralizing functionality of the antibodies transferred. Comparison of SARS-CoV-2-specific antibody effector functions in the serum and breastmilk (Figures 2A, 2B, and S3) pointed to significantly greater functionality of antibodies capable of inducing more robust levels of antibody-dependent cellular monocyte phagocytosis (ADCP), antibody-dependent neutrophil phagocytosis (ADNP), antibody-dependent NK cell activation (ADNKA) (degranulation/CD107a and chemokine secretion/Macrophage Inflammatory Protein-1β or MIP-1β), antibody-dependent complement deposition (ADCD), and neutralization (NT50) in the serum (Figure 2A). Conversely, limited transfer of functional antibodies was observed in the breastmilk (Figures 2B and 2C). Although all functional antibodies were lower in breastmilk compared to serum (breastmilk/serum ratio below 1; Figure 2C), neutralizing antibodies and neutrophil phagocytosing antibodies (ADNP) had higher transfer ratios into breastmilk compared to other functional antibodies (Figure 2C). Strikingly, breastmilk had limited NK-cell activating functions, which are known to transfer preferentially across the placenta (Jennewein et al., 2019), suggesting strict functional antibody selection into the breastmilk, potentially aimed at limiting inflammatory antibodies to the neonatal gut. Interestingly, whereas ADNP can be driven by IgG or IgA and neutralization can be driven by any antibody isotype, ADNKA is only induced by IgG, suggesting omission of highly inflammatory IgG from breastmilk in natural SARS-CoV-2 infection. Overall, these data demonstrate functional selection of antibodies into the breastmilk.

### Predictors of relative antibody abundance in breastmilk

**Figure 3:**
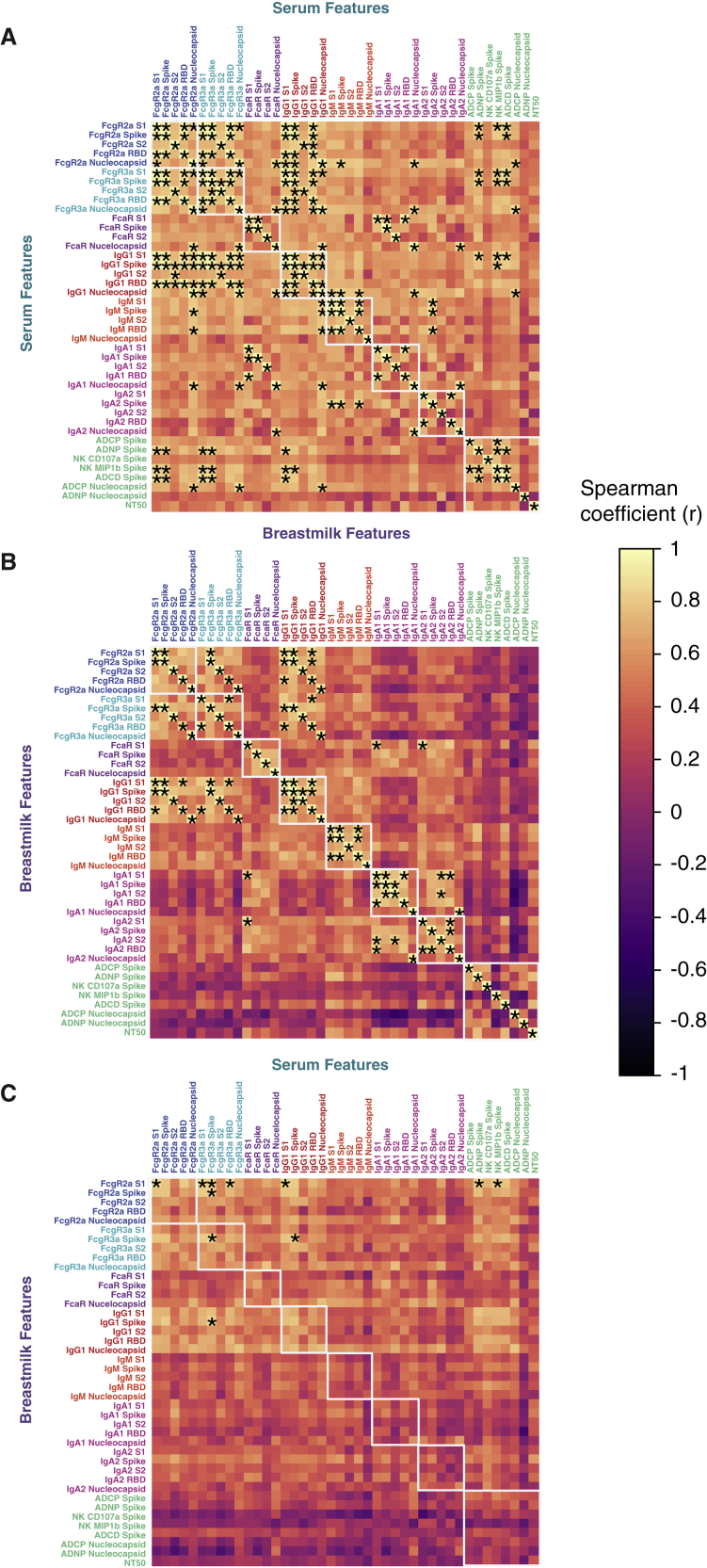
Correlation of antibody features in serum and breastmilk The heatmaps illustrate the spearman correlations between SARS-CoV-2 antibody features measured within the serum (A) and the breastmilk (B), as well as between serum and breastmilk features (C). Lighter coloration indicates a more positive correlation coefficient (r), while darker coloration indicates a more negative correlation coefficient. Significance was determined by a p < 0.05 after Bonferroni multiple hypothesis correction and is indicated by an asterisk.

To further explore the relationship between serum and breastmilk antibody profiles of SARS-CoV-2-infected mothers, correlations within and between biofluids were computed. Maternal serum antibody titers and FcR-binding levels were positively correlated across isotypes and FcR-binding antibodies (Figure 3A), as previously noted (Atyeo et al., 2021), highlighting a coordinated serum response elicited following infection. There was significantly less correlation across subclasses and isotypes in the breastmilk, with the exception of IgG1 correlations with FcR binding (Figure 3B). Although not statistically significant, weak negative trends were observed between antibody levels and immune functionality in breastmilk (Figure 3B). Together, these data suggest that the coordinated antibody signature in maternal serum is not conserved in the breastmilk.

Few statistically significant correlations were observed across serum and breastmilk (Figure 3C). Overall, most breastmilk features were weakly positively correlated with serum features. Despite lower relative abundance of IgG in breastmilk, the only strong positive relationships between serum and breastmilk were between IgG titers, IgG-binding FcRs (FcγR2a and FcγR3a), and antibody-driven neutrophil and NK cell function. The lack of significant correlation between maternal IgA titers and breastmilk IgA titers and functions suggests that breastmilk IgA may not be derived from maternal serum IgA but could instead be derived from mucosal plasma cells that may populate breastmilk in a selective manner (Wilson and Butcher, 2004). Moreover, consistent with the within-breastmilk analyses, the functional response in breastmilk was weakly negatively correlated with serum antibody titers (Figure 3C). These data suggest that, although functional antibodies are largely excluded from the breastmilk (Figure 2B), certain functional antibodies from the serum are linked to increased FcR binding in the breastmilk, pointing to a potential mechanism of selective functional antibody transfer to the breastmilk.

### Disease severity impacts the quality of breastmilk antibody

**Figure 4:**
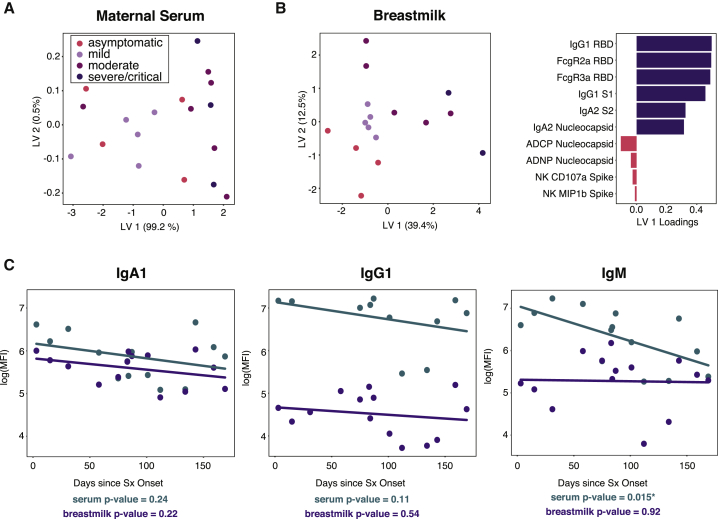
Contribution of disease severity and time since symptom onset on breastmilk antibody transfer (A) The scores plot of a partial least-squares regression (PLSR) model built on serum-derived antibody features with maternal disease severity, based on NIH criteria, as the outcome variable. (B) PLSR model built using breastmilk-derived antibody features with disease severity as the outcome variable. The dot plot (left) shows the scores of each sample, with each sample indicated by a dot and the color representing disease severity. The bar plot (right) illustrates the loadings of the features selected via Elastic Net on latent variable 1 (LV1), with the color indicating whether the feature was enriched in patients with milder (pink) or more severe (purple) COVID-19 symptoms. (C) Linear regression models fitting the relationship between days since symptom (Sx) onset and IgA1, IgG1, and IgM antibody titers in breastmilk (purple) and serum (teal). p values are reported below each plot.

Mounting evidence points to more robust humoral immune responses in the setting of more severe disease (Rijkers et al., 2020; Zohar et al., 2020). Thus, to investigate whether COVID-19 severity contributed to the overall level and function of antibodies in SARS-CoV-2-infected mothers and their breastmilk, we next classified the women into four groups based on NIH disease severity criteria (NIH, 2021). Differentiation based on disease severity was not observed in maternal serum SARS-CoV-2-specific antibody functional profiles based on disease severity (Figures 4A and S4A). However, breastmilk SARS-CoV-2-specific antibody profiles clustered more distinctly based on disease severity (Figures 4B and S4B). Women with more severe COVID-19 transferred enhanced levels of both FcR binding IgG and IgA antibodies against several SARS-CoV-2 specificities in breastmilk, whereas individuals with less severe disease transferred higher levels of functional antibodies, namely NK cell-activating (MIP-1β and CD107a) and nucleocapsid-specific ADNP- and ADCP-inducing antibodies (Figures 4B and S4C). These data indicate functional selection of antibodies that tracks with disease severity, suggesting that mothers with more severe disease, and potentially more inflammatory profiles in their serum, transfer higher titers of less functional antibodies into breastmilk.

### Time since symptom onset impacts antibody transfer into breastmilk

Lastly, emerging kinetic analyses of antibody responses following COVID-19 reveal early, near-simultaneous production of IgM, IgA, and IgG (Iyer et al., 2020), with subsequent decline of systemic IgM and IgA (Iyer et al., 2020; Sun et al., 2020). Thus, we sought to characterize the COVID-19 antibody response in maternal serum and breastmilk as a function of time to begin to understand time-dependent relationships across the compartments. IgA responses declined only slightly across both the serum and the breastmilk over time after symptom onset (Figure 4C), highlighting the steady production and transfer of this isotype. Similarly, serum-derived IgG1 weakly decreased over time, but spike-specific IgG1 antibodies were transferred weakly but steadily to breastmilk, irrespective of time from infection. Strikingly, IgM titers waned significantly over time in the peripheral circulation, as expected, but breastmilk transfer remained robust over time from symptom onset (Figure 4C). These trends mark highly stable IgM, IgA, and IgG transfer to the breastmilk, despite loss of IgM in the serum, irrespective of time from symptom onset. The persistent IgM response in the breastmilk may reflect the continued production of secretory IgM that may uniquely populate breastmilk during lactation, even after the serum IgM response to natural infection is lost, highlighting distinct humoral mechanisms at play following childbirth to protect infants from infection.

## Discussion

Despite the rapid emergence of highly protective COVID-19 vaccines globally, infants will likely be among the last to receive vaccines, due to the enhanced safety concerns related to vaccinating this population. However, infants can be protected against disease via antibody transfer from their mothers through the placenta and through breastmilk. Although significant progress has been made in deciphering the selectivity of antibody transfer across the placenta, less is known about the mechanisms by which IgG is transferred from blood to breastmilk, and IgA and IgM are transferred from mucosal plasma cells and plasmablasts into breastmilk. Such transfer occurs following birth to promote mucosal immunity against pathogens, including SARS-CoV-2. While the transfer of neutralizing antibodies into breastmilk is critical (Fox et al., 2020; Pace et al., 2020), antibody functions beyond neutralization are also key to protective immunity (Excler et al., 2014; Lu et al., 2018; Mabuka et al., 2012). Deep humoral profiling identified the expected selective transfer of persistent IgA and IgM to breastmilk, with reduced and functionally restricted but persistent IgG transfer. Surprisingly, cytotoxic antibody functions were largely excluded from the breastmilk, although neutrophil phagocytosis and neutralization were preferentially transferred in relation to the level of these antibody functions in maternal blood. These data point to a previously unappreciated selection of antibodies across to breastmilk that may provide critical insights for the design of next-generation vaccines or therapeutics to protect mothers and their infants after birth.

This study systematically and broadly measured antibody isotypes, FcR binding, and antibody function in breastmilk following COVID-19. Breastmilk contained notable ADCP, ADNP, and ADCD activity. Surprisingly, we detected limited NK activity in the breastmilk. Whether this is a SARS-CoV-2-specific phenomenon has yet to be determined. Previous studies have shown that breastmilk antibodies can drive antibody-dependent cellular cytotoxicity (ADCC) via NK cells against HIV, linked to reduced infection rates in the infants (Mabuka et al., 2012). However, HIV is a chronic infection, marked by extraordinarily high levels of antibodies in infected women, even when on antiretroviral therapy. Thus, whether NK cell functions are transferred in the setting of chronic exposure but may be excluded following recent infection remains to be determined. High levels of ADNP-inducing antibodies were transferred against spike and nucleocapsid, suggesting that there may be preferential transfer of antibodies more apt to confer protection at mucosal barriers in response to infection. Given the potential immunopathologic activity of NK cell-activating antibodies (Maucourant et al., 2020), compared to the less cytopathic role of neutrophil-eliciting antibodies that have been linked to resolution of severe COVID-19 (Atyeo et al., 2020), it is plausible that this selective transfer of functionality represents a critical evolutionary mechanism to provide infants with appropriately protective and not destructive functional antibodies in the mucosa. However, some studies have suggested a hyper-inflammatory role for IgA able to recruit neutrophils at mucosal surfaces, particularly in autoimmune diseases (Breedveld and van Egmond, 2019). Therefore, further research must be done to understand the role of functional antibodies both in breastmilk and at mucosal surfaces.

Whether immune transfer through breastmilk will remain stable for months after infection remains incompletely understood. Longitudinal studies on breastmilk composition have demonstrated that the concentration of antibodies decreases in mature milk compared to colostrum (Goldman et al., 1982; Goonatilleke et al., 2019). However, how antibody quality and functionality changes over the months following birth is unknown. Although emerging data point to a critical role for IgA in regulating commensalization (Palm et al., 2014; Rogier et al., 2014), the data here argue for long persistence of natural infection-induced transfer of all antibody isotypes, dominated by both IgA and IgM antibodies via breastmilk to the infant. Whether these antibodies are drawn from recruited plasma cells seeded within the lung remains unclear but may have important implications for vaccines that will not directly lead to the induction of lung-resident plasma cells.

Despite the relatively low incidence of neonatal and infant SARS-CoV-2 infection, this population is more likely to require hospitalization compared to other pediatric groups (Kim et al., 2020). Because vaccines will likely be tested last in this population, young children will remain vulnerable long after vaccines have rolled out across the globe. Moreover, given the potential for this virus to remain endemic in our population, an understanding of mechanisms to protect infants is urgently needed (Lavine et al., 2021; Shaman and Galanti, 2020). These data illustrate biased transfer of antibody isotypes that can be coupled to a secretory chain, IgM and IgA, into breastmilk following natural infection, as well as the selection of antibodies with particular functional capabilities. Emerging data from vaccinated pregnant and lactating women suggest that vaccine-induced transfer may be altered due to the extraordinarily high levels of IgG antibodies induced by the current Emergency Use Authorization-approved mRNA vaccines, providing infants with both robust IgA and IgG immunity (Gray et al., 2021) that may be able to confer enhanced immunity compared to natural infection. Studies are needed to understand the durability of antibody transfer following both natural and vaccine-induced protection to guide vaccine design and deployment in the future for this uniquely vulnerable population.

### Limitations

This study focused on immune profiling of colostrum to ensure uniform comparison of milk. Because only small volumes were available at the time of collection, 1 to 2 days postpartum, the study was unable to examine other antibody specificities or how this immune transfer may change over time with the change in milk over the course of lactation. However, future studies able to collect milk from birth throughout the first few months of life may have the opportunity to yield additional insights into both the persistence and changes in quality of SARS-CoV-2-specific antibody transfer over time. Moreover, comparison to other pathogen-specific antibodies may provide new insights into the mechanisms by which antibodies confer protection in the mucosa of neonates and provide an opportunity to deconvolute the rules of antibody transfer to breastmilk to guide next-generation vaccine design.

## STAR★Methods

### Key resources table

**Table:** 

REAGENT or RESOURCE	SOURCE	IDENTIFIER
**Antibodies**		

anti-CD66b-Pacific blue	BioLegend	CAT # 305112
APC-Cy7 Mouse Anti-Human CD16	BD Biosciences	CAT # 557758 RRID:AB_396853
CD56 PE-Cy7 Mouse Anti-Human CD56	BD Biosciences	CAT # 557747
PE MIP-1b Mouse anti-Human	BD Biosciences	CAT # 550078 RRID:AB_393549
Pacific Blue Mouse Anti-Human CD3	BD Biosciences	CAT # 558117 RRID:AB_1595437
FITC Goat IgG anti-C3	MP Biomedicals	CAT # 855385
Mouse Anti-Human IgG1-Fc PE	Southern Biotech	CAT # 9054-09
Mouse Anti-Human IgG2-Fc PE	Southern Biotech	CAT # 9060-09
Mouse Anti-Human IgG3-Hinge PE	Southern Biotech	CAT # 9210-09
Mouse Anti-Human IgG4-Fc PE	Southern Biotech	CAT # 9200-09
Mouse Anti-Human IgA1-Fc PE	Southern Biotech	CAT # 9130-09
Mouse Anti-Human IgM-Fc PE	Southern Biotech	CAT # 9020-09

**Bacterial and virus strains**		

SARS-CoV-2-S pseudovirus with a luciferase reporter	This paper	N/A

**Chemicals, peptides, and recombinant proteins**		

SARS-CoV-2 S	Lake Pharma	N/A
SARS-CoV-2 RBD	Sino Biological	CAT # 40592-V08H
SARS-CoV-2 N	Aalto Bio Reagents	CAT # CK 6404-b
SARS-CoV-2 S1	Sino Biological	Cat # 40591-V08H
SARS-CoV-2 S2	Sino Biological	CAT # 40590-V08B
A/Michigan/45/2015 (H1N1)	Immunetech	CAT # IT-003-00105ΔTMp
B/Phuket/3073/2013	Immunetech	CAT # IT-003-B11ΔTMp
A/Singapore/INFIMH-16-0019/2016	Immunetech	CAT # IT-003-00434ΔTMp
Human Fc receptors	Produced at the Duke Human Vaccine Institute, {Boesch, 2014 #15}	N/A
Streptavidin-R-Phycoerythrin	Prozyme	CAT # PJ31S
FIX&Perm Cell Permeabilization Kit	Life Tech	CAT # GAS001S100
		CAT # GAS002S100
Human IL-15 Recombinant Protein, eBioscience	ThermoFisher Scientific	CAT # BMA31
Brefeldin A	Sigma Aldrich	CAT # B7651
GolgiStop	BD Biosciences	CAT # 554724

**Critical commercial assays**		

BirA-500: BirA biotin-protein ligase standard reaction kit	Avidity	CAT # BirA500
RosetteSep Human NK Cell Enrichment Cocktail	Stem Cell Technologies	CAT # 15065
Steady-Glo Luciferase Assay	Promega	CAT # E2510

**Deposited data**		

Generated Code	This paper	https://doi.org/10.5281/zenodo.5567701

**Experimental models: cell lines**		

THP-1 Cells	ATCC	CAT # TIB-202 RRID: CVCL_0006

**Software and algorithms**		

GraphPad Prism	GraphPad	https://www.graphpad.com/scientificsoftware/prism/
Intellicyt ForeCyt Software	Sartorious	https://intellicyt.com/products/software/
R programming language	Version 4.0.0	https://www.r-project.org/

**Other**		

FluoSpheres NeutrAvidin-Labeled Microspheres, 1.0 μm, yellow-green fluorescent (505/515), 1% solids	Invitrogen	CAT # F8776
FluoSpheres NeutrAvidin-Labeled Microspheres, 1.0 μm, red fluorescent (505/515), 1% solids	Invitrogen	CAT # F8775
MagPlex microspheres	Luminex corporation	CAT # MC12001-01, MCI12040-01, MCI10077-01

### Resource availability

#### Lead contact

Further information and requests for resources and reagents should be directed to and will be fulfilled by the Lead Contact, Galit Alter (galter@partners.org).

#### Materials availability

This study did not generate new unique reagents.

### Experimental model and subject details

#### Sample Cohort

Maternal serum and breastmilk were collected from 20 lactating women who were previously infected during pregnancy with SARS-CoV-2 and 26 uninfected lactating women who were contemporaneously enrolled (Table 1). After quality control, one sample was removed from analyses. All SARS-CoV-2 + individuals were tested either at the time of symptom onset or at the delivery admission (for asymptomatic positives) by nasopharyngeal swab and real-time reverse-transcriptase polymerase chain reaction (RT-PCR). The SARS-CoV-2 negative control population was defined as those never known to be positive for SARS-CoV-2 at any time in pregnancy and were asymptomatic and tested negative for SARS-CoV-2 by nasopharyngeal swab and RT-PCR at delivery. Maternal serum was collected at the time of delivery. Breastmilk samples were collected 1-3 days after delivery. Samples were collected at Massachusetts General Hospital (MGH), Brigham and Women’s Hospital (BWH) and Beth Israel Deaconess Medical Center (BIDMC). All enrollees provided informed consent. This study was approved by the MGH-BWH Institutional Review Board and the BIDMC Institutional Review Board.

#### Cell Lines

THP-1 cells were purchased from ATCC (ATCC® TIB-202), were grown at 37°C, 5% CO2 and were maintained in RPMI with 10% fetal bovine serum, penicillin/streptomycin, L-glutamine, HEPES, and beta-mercaptoethanol.

#### Primary Immune Cells

Fresh peripheral blood was collected at MGH and the Ragon Institute from healthy volunteers. All volunteers gave written consent, were over 18, and were deindentified prior to blood processing. Neutrophils isolated from peripheral blood were maintained at 37°C, 5% CO2 in RPMI with 10% fetal bovine serum, L-glutamine, HEPES, and penicillin/streptomycin. Human NK cells isolated from peripheral blood were maintained at 37°C, 5% CO2 in RPMI with 10% fetal bovine serum, L-glutamine, HEPES, penicillin/streptomycin and IL-15 for the duration of the assay. The study was approved by the MGH Institutional Review Board.

### Method details

#### Isotype and FcR-binding measurements

A multiplexed luminex assay was used to measure antigen-specific isotypes and FcR-binding, as previously described (Brown et al., 2017). Briefly, antigens were covalently linked to carboxyl-modified Magplex © Luminex beads using Sulfo-NHS (Pierce) and EDC (Thermo Fisher). Antigens used for this assay were SARS-CoV-2 RBD (kindly provided by Aaron Schmidt), SARS-CoV-2 S (kindly provided by Eric Fischer), SARS-CoV-2 N (Aalto Bio Reagents). SARS-CoV-2 S1 (Sino Biological), and SARS-CoV-2 S2 (Sino Biological). Antigen-coupled beads were blocked with PBS-TBN, resuspended in PBS, and maintained at 4°C.

Immune complexes were formed by adding antigen coupled beads to appropriately diluted serum or breastmilk supernatant. Plates were then incubated overnight at 4°C, shaking at 700 rpm. The next day, plates were washed in assay buffer (0.1% BSA, 0.02% Tween in PBS). To detect antigen-specific isotypes, immune complexes were stained with PE-coupled mouse anti-human IgG1, IgA1, IgA2, or IgM (Southern Biotech). To detect FcR-binding, Avi-Tagged FcRs (Duke Human Vaccine Institute) were biotinylated using a BirA500 kit (Avidity). The biotinylated FcRs were then labeled with streptavidin-PE and added to the immune complexes. Fluorescence was acquired using an iQue (Intellicyt). Antigen-specific isotype titer and FcR-binding was reported as the median fluorescence intensity (MFI).

#### Antibody-dependent cellular phagocytosis (ADCP)

The ADCP assay was performed as previously described (Ackerman et al., 2011). SARS-CoV-2 spike (kindly provided by Eric Fischer) and nucleocapsid (Aalto Bioreagents) was biotinylated using Sulfo-NHS-LC-LC-biotin (Thermo Fisher), desolated using Zeba columns (Thermo Fisher), and coupled to yellow-green Neutravidin beads (Invitrogen) for 2 hours at 37°C or overnight at 4°C. Coupled beads were washed twice in 0.01% BSA in PBS and resuspended at 10 ug/mL for use in the assay. Immune complexes were formed by adding coupled beads to 96-well plates with equal volume of diluted serum (1:100) or diluted breastmilk (1:10). Immune complexes were incubated for two hours at 37°C. After the incubation, the immune complexes were washed, and THP-1 cells were added to the immune complexes at 1.25x10ˆ5 cells/mL. Cells were incubated with the immune complexes overnight at 37°C. The next day, the cells were fixed in 4% PFA. Fluorescence was acquired using an iQue (Intellicyt) and analyzed using Forecyt software. A Phago score was determined using the following formula: (percentage of bead-positive cells) x (GeoMean of MFI of bead-positive cells)/10,000

#### Antibody-dependent neutrophil phagocytosis (ADNP)

The ADNP assay was performed as described (Karsten et al., 2019). Spike and nucleocapsid biotinylation, bead coupling, and immune complex formation was performed as described for ADCP. Leukocytes were isolated from fresh peripheral blood from healthy donors (Ragon Institute) by ammonium-chloride potassium (ACK) lysis. After immune complex incubation, immune complexes were washed and leukocytes were added at a concentration of 2.5 × 10ˆ5 cells/mL. Cells and immune complexes were incubated for 1 hour at 37°C. Following incubation, neutrophils were stained using anti-CD66b Pacblue (Biolegend). Cells were fixed with 4% PFA. Fluorescence was acquired as described for ADCP.

#### Antibody-dependent complement deposition (ADCD)

The ADCD assay was performed as previously described (Fischinger et al., 2019). Spike biotinylation, bead coupling, and immune complex formation was performed as described for ADCP, using red Neutravidin beads (Invitrogen) and 1:10 dilution of serum and 1:1 dilution of breastmilk. Following immune complex formation, plates were washed and guinea pig complement (Cedarlane) diluted in gelatin veronal buffer supplemented with calcium and magnesium (Boston BioProducts) was added. Plates were incubated for 20 minutes at 37°C. Plates were washed twice with 15mM EDTA in PBS and C3-deposition was detected by staining with anti-C3 FITC (MPbio). Fluorescence was acquired using an iQue (Intellicyt) and C3-deposition is reported as the median fluorescence intensity of FITC.

#### Antibody-dependent NK cell activation (ADNKA)

ELISA plates were coated with 2 ug/mL of spike, incubated for 2 hours at 37°C, washed three times with PBS and blocked overnight at 4°C in 5% BSA in PBS. Human NK cells were isolated from peripheral blood (MGH Blood Bank) using RosetteSep kit (Stem Cell Technologies) followed by Ficoll separation to isolate cells. NK cells were maintained overnight at 37°C in RPMI media with 10% fetal bovine serum, L-glutamine, HEPES, penicillin/streptomycin and IL-15. Blocked plates were washed three times with PBS, and diluted serum (1:50) and diluted breastmilk (1:5) were added to the coated ELISA plates. Plates were incubated for 2 hours at 37°C. After the incubation, plates were washed three times with PBS, and NK cells were added at a concentration of 2.5 × 10ˆ5 cells/mL in media supplemented with GolgiStop (BD), Brefeldin A (BFA, Sigma Aldrich) and anti-CD107a PE-Cy5 (BD) and were incubated for 5 hours at 37°C. Following the incubation, NK cells were stained for surface markers with anti-CD3 PacBlue (BD), anti-CD16 APC-Cy5 (BD), and anti-CD56 PE-Cy7 (BD). After staining, cells were fixed using the FIX&PERM A/B kit (Life Tech) and stained for MIP-1b (anti-MIP-1b PE, BD). Fluorescence was acquired using an iQue (Intellicyt). NK cells were gated as CD56+/CD16+/CD3- and NK cells activity was determined as the percentage of NK cells that were positive for CD107a and MIP-1b.

### Quantification and statistical analysis

#### Univariate Analysis

Univariate data was visualized and analyzed using Graphpad software, version 8.0. The data is plotted as the average of two replicates. Breastmilk data was dilution corrected. Transfer ratios were calculated by dividing breastmilk data by serum data for a particular patient. Spearman correlations between features were calculated using the 'corrplot' package (version 0.90) in R (version 4.0.0). Bonferroni multiple hypothesis correction was performed to determine significant correlations.

#### Multivariate Analyses

Multivariate analyses were performed in R (version 4.0.0). The raw luminex data was log transformed, centered and scaled. Partial least square regression (PLSR) was performed to regress luminex features on severity scores for maternal infection (Figure 4). PLSR models were generated with the ‘ropls’ Bioconductor package. Prior to PLSR, features were reduced using Elastic Net variable regularization and selection to avoid feature redundancy and overfitting (Zou and Hastie, 2005). Utilizing the ‘caret’ package, 100 trials of Elastic Net were run, selecting features present in 35% or more of the Elastic Net models to be included in the final PLSR. PLSR models were validated using leave-one-out cross validation (19 trials). A linear regression was performed to correlate the predicted ‘y’ values (or antibody titer) with the actual ‘y’ values, reporting the slope and standard deviation. A perfect model would have a slope of 1.

## Data Availability

The dataset generated during this study is available upon reasonable request. Code for the multivariate analyses can be found at https://github.com/Lauffenburger-Lab or zenodo (DOI 10.5281/zenodo.5567701). Any additional information required to reanalyze the data reported in this paper is available from the lead contact upon request.

## Supplemental information

Document S1. Figures S1–S4

Document S2. Article plus supplemental information
