# Supplementary material for: Selective functional antibody transfer into the breastmilk after SARS-CoV-2 infection
Source: Cell Rep. 2021 Oct 22;37(6):109959. doi: 10.1016/j.celrep.2021.109959 (PMC8531199; doi:10.1016/j.celrep.2021.109959)
Supplement: Document S1. Figures S1–S4 [file mmc1.pdf]

Cell Reports, Volume *37*

Supplemental information

Selective functional antibody transfer

into the breastmilk after SARS-CoV-2 infection

Krista M. Pullen, Caroline Atyeo, Ai-Ris Y. Collier, Kathryn J. Gray, Mandy B. Belfort, Douglas A. Lauffenburger, Andrea G. Edlow, and Galit Alter

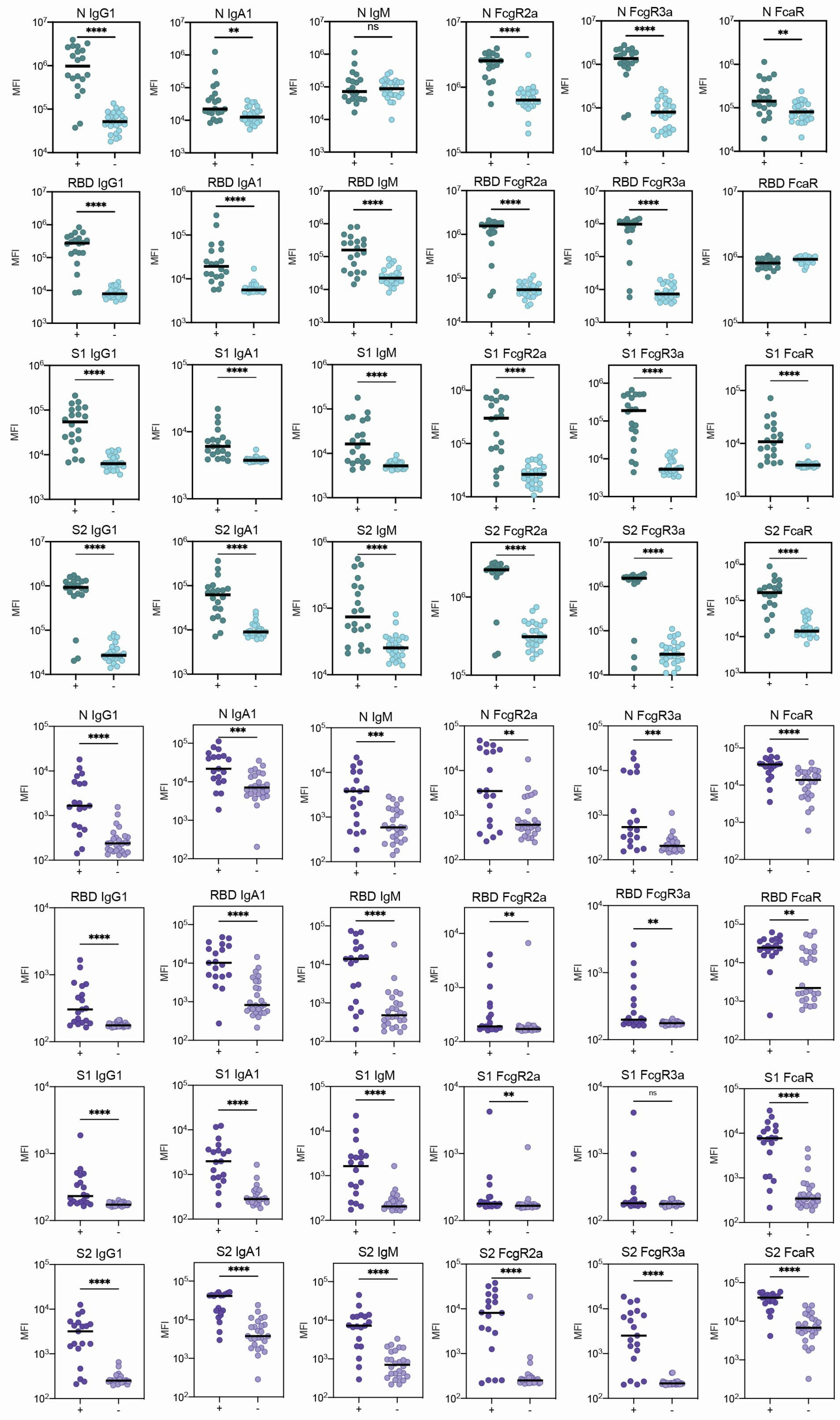

N IgG1 ****
N IgA1 **
N IgM ns
N FcgR2a ****
N FcgR3a ****
N FcaR **
RBD IgG1 ****
RBD IgA1 ****
RBD IgM ****
RBD FcgR2a ****
RBD FcgR3a ****
RBD FcaR
S1 IgG1 ****
S1 IgA1 ****
S1 IgM ****
S1 FcgR2a ****
S1 FcgR3a ****
S1 FcaR ****
S2 IgG1 ****
S2 IgA1 ****
S2 IgM ****
S2 FcgR2a ****
S2 FcgR3a ****
S2 FcaR ****
N IgG1 ****
N IgA1 ***
N IgM ***
N FcgR2a **
N FcgR3a ***
N FcaR ****
RBD IgG1 ****
RBD IgA1 ****
RBD IgM ****
RBD FcgR2a **
RBD FcgR3a **
RBD FcaR **
S1 IgG1 ****
S1 IgA1 ****
S1 IgM ****
S1 FcgR2a **
S1 FcgR3a ns
S1 FcaR ****
S2 IgG1 ****
S2 IgA1 ****
S2 IgM ****
S2 FcgR2a ****
S2 FcgR3a ****
S2 FcaR ****
MFI
+
-

Figure S1. SARS-CoV-2-infection results in the induction of SARS-CoV-2 antibodies against multiple specificities in serum and breastmilk.
The dot plots show the nucleocapsid (N), receptor-binding domain (RBD), S1 and S2 antibody response in serum (top, teal) and breastmilk (bottom, purple). Significance was determined by Mann-Whitney test, * $p<0.05$, ** $p<0.01$, *** $p<0.001$, **** $p<0.0001$. The data represents the average of two replicates. Related to Figure 1.

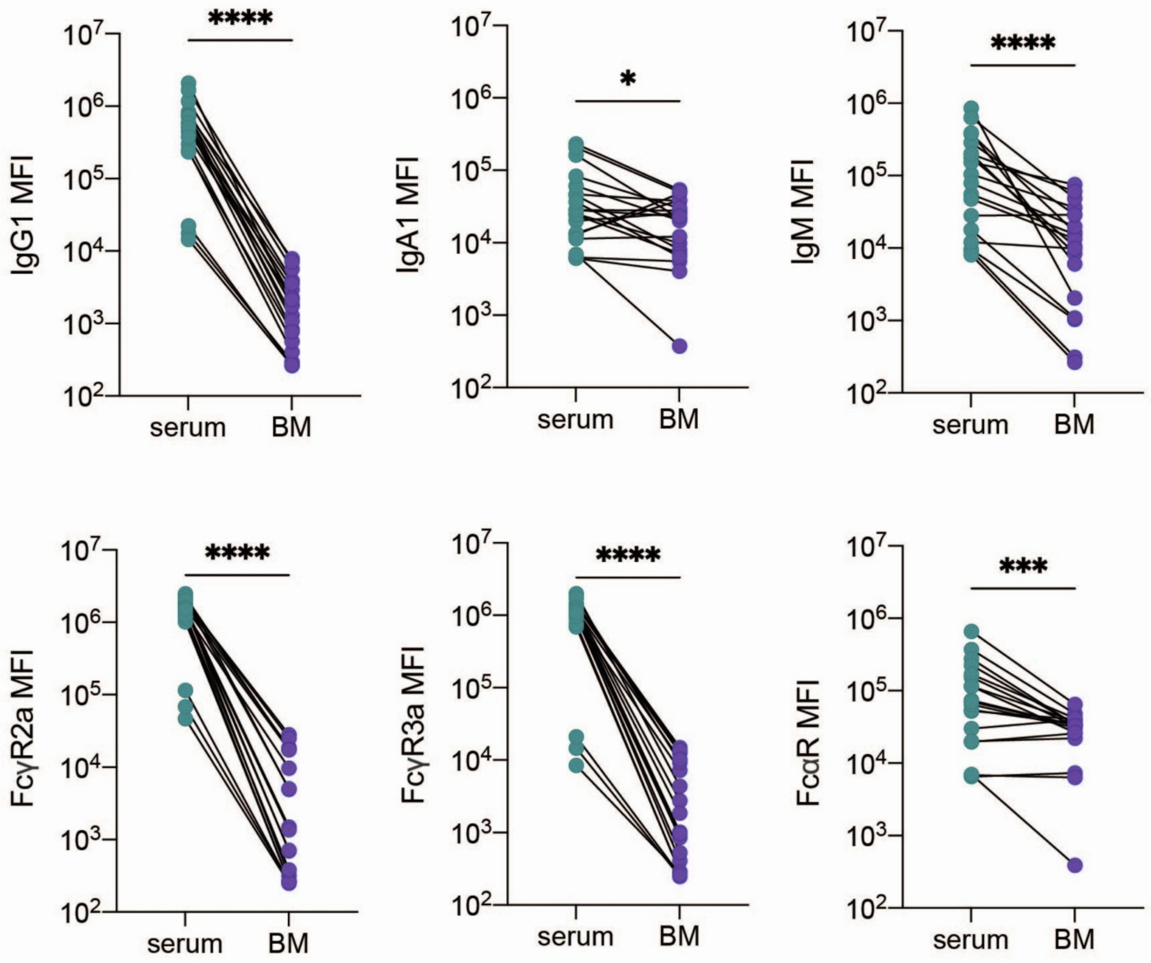

Figure S2. SARS-CoV-2 infection results in a higher antibody response in serum than in breastmilk.
The dot plots show the relative IgG1, IgM, and IgA titer and FcgR2a, FcgR3a and FcaR binding in serum (teal) and breastmilk (purple) of individuals previously infected with SARS-CoV-2. Lines connect serum and breastmilk samples from the same individual. Significance was determined by a Wilcoxon matched pairs signed rank test, * $p<0.05$, ** $p<0.01$, *** $p<0.001$, **** $p<0.0001$. The data represents the average of two replicates. Related to Figure 1.

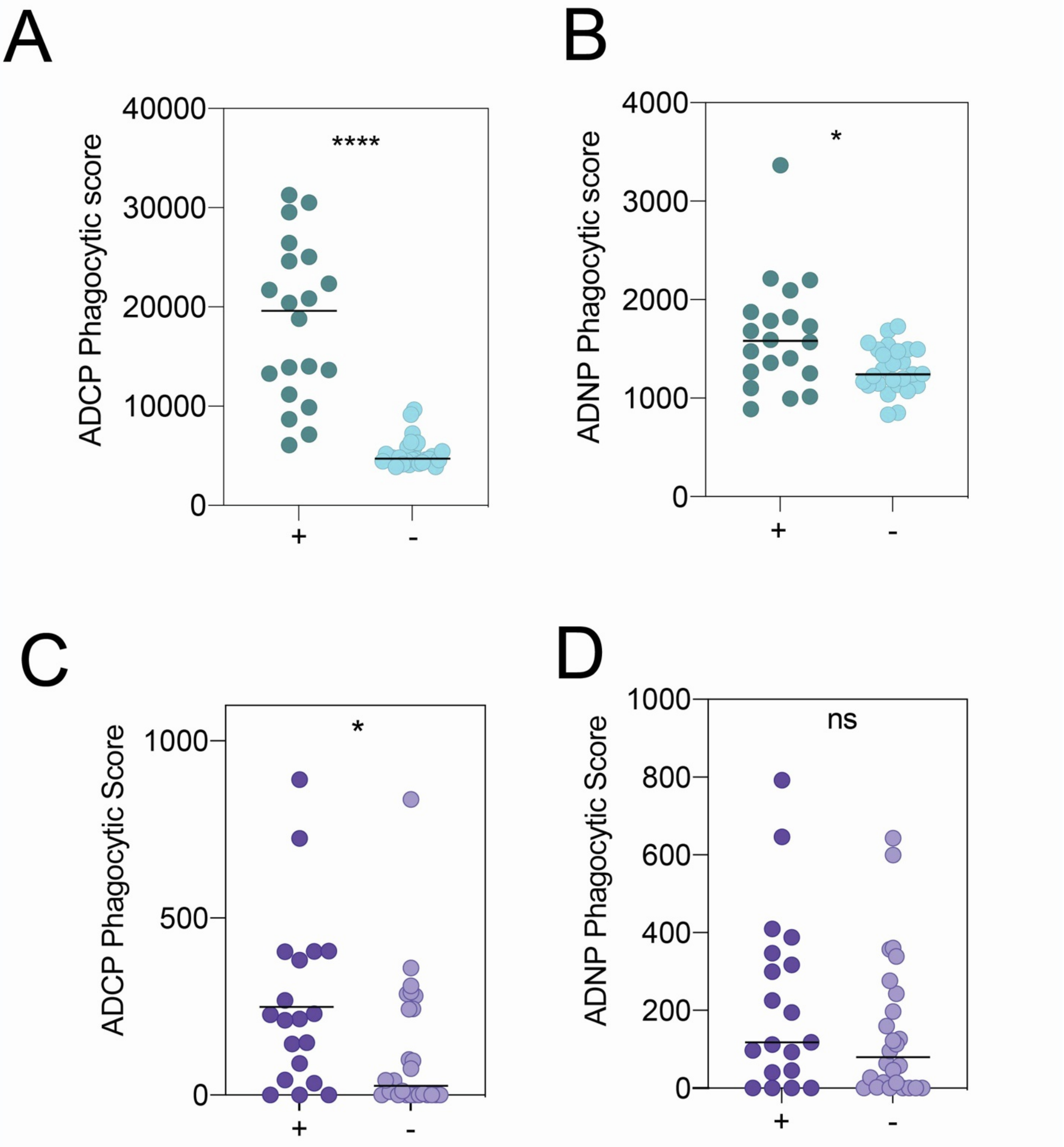

Figure S3. SARS-CoV-2 infection induces functional antibodies against Nucleocapsid in serum and breastmilk.

The dot plots show the ADCP and ADNP antibody response in serum (A and B) and breastmilk (C and D). Significance was determined by Mann-Whitney test, * $p<0.05$, ** $p<0.01$, *** $p<0.001$, **** $p<0.0001$. The data represents the average of two replicates (ADCP) or two donors (ADNP). Related to Figure 2.

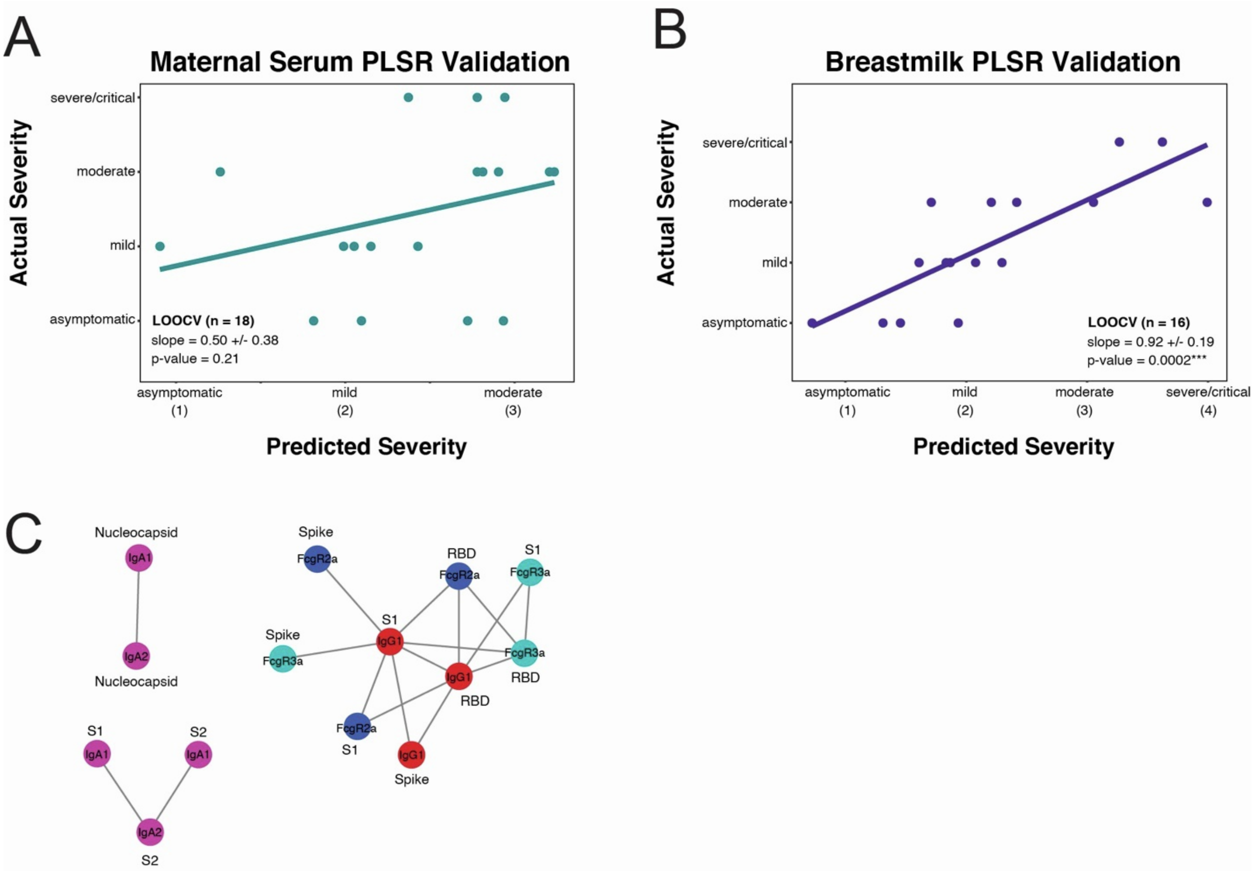

Figure S4. Model Validation and Correlates Predicting Disease Severity
(A) Leave-one-out cross validation (LOOCV) for the PLSR model in Figure 4A regressing maternal serum features onto disease severity. Each dot on the plot represents the predicted (x-axis) and actual (y-axis) severity labels for one serum sample. A line was fit to the plot with the categorical labels converted to numeric labels, as indicated in parentheses on the x-axis. The p-value for the fit was reported as 0.21. (B) LOOCV for the PLSR model in Figure 4B regressing breastmilk features onto disease severity. Each dot on the plot represents the predicted (x-axis) and actual (y-axis) severity labels for one breastmilk sample. A line was fit to the plot with the categorical labels converted to numeric labels, as indicated in parentheses on the x-axis. The p-value for the fit was reported as 0.0002. (C) Correlation network of the breastmilk features significantly correlated to the Elastic Net-selected features included in Figure 4B, in concordance with the correlation plots in Figure 3B.
